# Incidental case finding of a 19‐year‐old woman with Tuberous Sclerosis Complex: A step‐wise Multidisciplinary approach

**DOI:** 10.1002/ccr3.4933

**Published:** 2021-10-06

**Authors:** Sajjad Ali, Samahir Tariq Khan, Hamza Usman, Ali Hamza Khan, Zain Douba, Murtaza Ali

**Affiliations:** ^1^ Department of Medicine Ziauddin Medical University Karachi Pakistan; ^2^ Faculty of Medicine University of Aleppo Aleppo Syria; ^3^ Department of Medicine Dr. Ruth K.M. Pfau Civil Hospital Karachi Pakistan

**Keywords:** MRI, neurocutaneous, shagreen patch, tuberous sclerosis complex

## Abstract

Radiological imaging plays a vital role in clinically diagnosing TSC. TSC prognosis is largely determined by the severity and the extent of the systems affected by it. TSC patients are symptomatically managed, since no cure is present. Healthcare professionals must frequently check‐up TSC patients who have a lifelong disorder.

## Graphical abstract

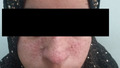

## 1 INTRODUCTION

Tuberous sclerosis complex (TSC) is a rare neurocutaneous condition characterized by the formation of benign tumors. We report an incidental case of 19‐year‐old woman with 3 major features for TSC with several minor features. Conclusion: TSC is a lifelong illness, where radiological imaging plays a vital role in clinical diagnosis. Although there is no cure, there is symptomatic therapy available.

Tuberous sclerosis complex (TSC) is conventionally an autosomal dominant disorder of neurocutaneous origin, defined as benign growths with multi‐organ involvement.^1^ These lesions range may present as angiofibromas or neurofibromas with frequently identifiable glial cell tumors in the brain cerebrum and/or retina.^2^ Mutations have been attributed to either of two genes, TSC1 and TSC2 which encode for tumor suppressor proteins. One in 10,000 live births may be affected by TSC with broad variations in clinical presentations. The TSC triad has been alternatively termed as EPILOIA, which originates from the three significant findings of the disease, that is, epilepsy (EPI), intellectual disability (LOI), and adenoma sebaceum (A).

The clinical criteria for diagnosing TSC are based on major and minor features of the disorder, with the presence of two major features or an amalgamation of one major and two minor features pertaining to a confirmed TSC diagnosis (Appendix S1). The authors here, bring forth an incidental case of TSC in a 19‐year‐old woman based on clinical and radiological analysis.

## 2 CASE HISTORY

A 19‐year‐old woman consulted the gastroenterology clinic in Dr. Ruth K.M. Pfau Civil Hospital Karachi, with primary complaints of a two‐month‐old shortness of breath and episodes of high‐grade fever since the past 3 weeks. This was vaguely preceded by intermittent episodes of diarrhea and diffuse abdominal pain for the last 10 months, concomitant with unexplained, and undocumented, weight loss. The patient reported an exacerbation of symptoms with increasing exertion. The fever lacked any signs of localized infection or acute systemic association. This was managed at home with regular antipyretics. The patient's past medical history is insignificant unless there have been numerous episodes of seizures since the age of six, with no continuous treatment acquired. All other aspects of development, including pre‐pubertal changes and menstrual health, have been unremarkable. The family history is positive for hypertensive disorders but none of the same accord.

**FIGURE 1:**
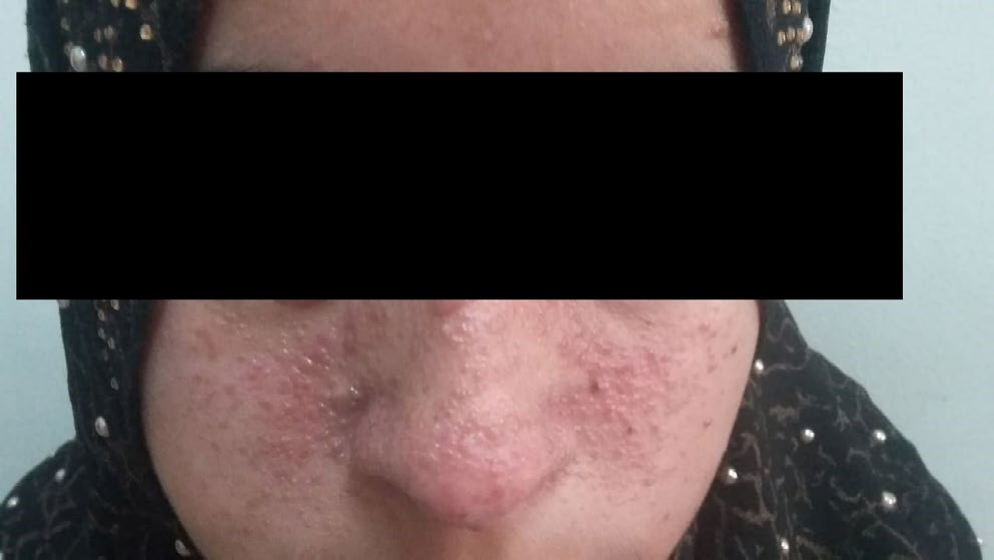
Angiofibroma lesion on the face

**FIGURE 2:**
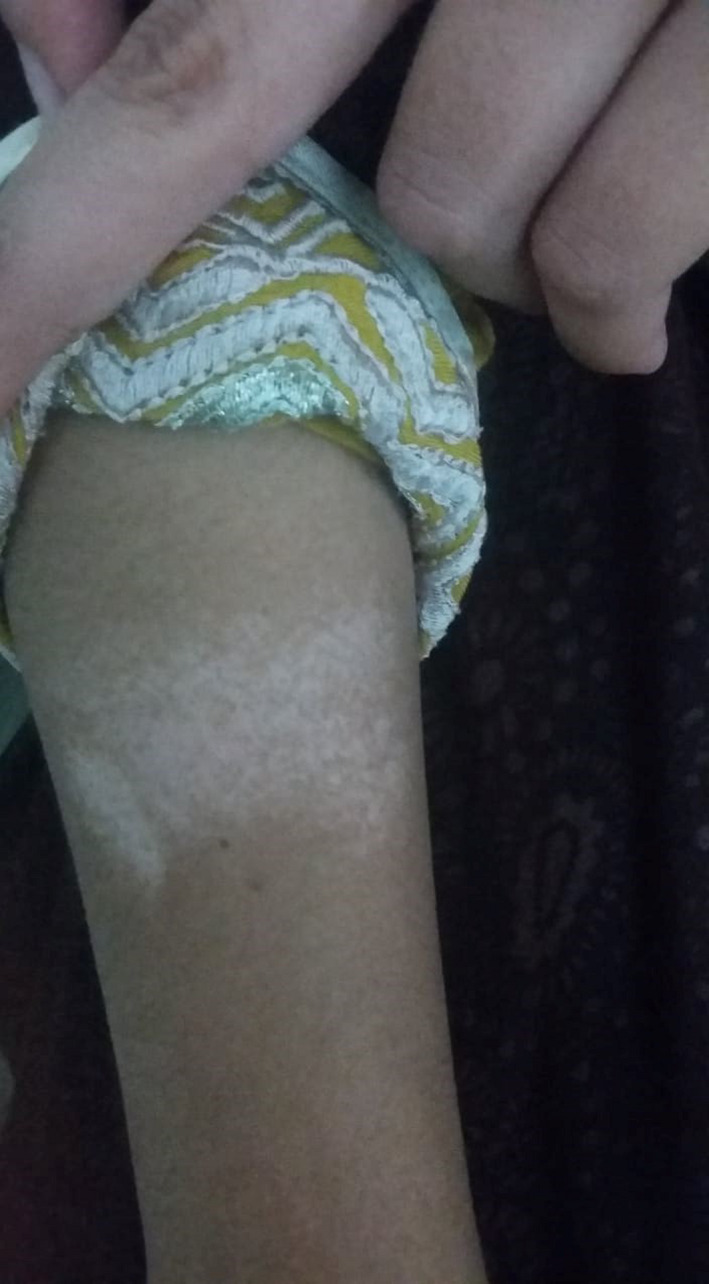
Hypopigmented lesion on the elbow

At presentation, she was febrile, tachycardic but hemodynamically stable. On examination, there was marked pallor, indicating positive anemic status. Grouped erythematous vesiculopapular lesions were noted on the face, asymmetrically spread over both cheeks, nose, and forehead (Figure 1). Several hypopigmented lesions could be detected, in the moderately lit clinic, on the extensor and flexor surfaces of both limbs and the truncal region (Figure 2).

The abdominal findings, however, showed ballotable kidneys. Considering the epidemiological causes of such a presentation, disseminated tuberculosis topped the list of differential diagnoses, followed by inflammatory bowel disease (specifically Crohn's disease), celiac disease, connective tissue disorders, neurocutaneous disorders, and polycystic kidney disease.

To rule out potential diagnoses, extensive investigations were carried out (Table 1). A CBC profile confirmed low hemoglobin, with an elevated MCV and MCH. In‐depth reasoning of anemia led to reporting of decreased serum B12 levels and increased RBC folate levels. Other cell lines were also deranged with a high leukocyte count and marked neutrophilia. Based on the results, further evaluation of inflammatory markers, that is, ESR and C‐reactive protein, both showed abnormally elevated serum levels. Moreover, the urinalysis report was positive for protein, red blood cell casts, leukocytes, and pus cells. A detailed stool culture was also carried out for evaluating infectious causes of diarrhea but the sample was only positive for mucus. Further investigations included anti‐transglutaminase IgA and IgG levels, both of which were low, denying the suspicion of celiac disease.

**TABLE 1:** Baseline investigations of the patient on the day of admission

Test Name	Result	Normal ranges
Complete blood count		
Hemoglobin (g/dl)	5.8	11–14.5
MCV (fL)	125	78.1–95.3
MCH (pg)	37.9	25.3–31.7
MCHC (Gm/dl)	32.5	30.3–34.4
TLC (counts/µl)	21000	4600–10800
Neutrophils (%)	83	34.9–76.2
Lymphocytes (%)	11	17.5–45
Platelet (counts/µl)	343000	154000–433000
Biochemical profile		
Blood Urea Nitrogen (mg/dl)	18	8–21
Creatinine (mg/dl)	0.62	0.8–1.3
Sodium (mEq/L)	134	135–145
Potassium (mEq/L)	3.7	3.5–5
Chloride (mEq/L)	99	95–105
Calcium (mg/dl)	8.4	8.5–10.2
Magnesium (mg/dl)	1.4	1.5–2
Phosphorous (mg/dl)	3.9	3.0–4.5
RBS (mg/dl)	114	<140
Hba1C	5.5%	<5.7%
Liver function test		
Total Bilirubin (mg/dl)	0.31	0.3–1.0
SGPT (U/L)	19	7–56
Alkaline Phosphatase (U/L)	299	50–100
SGOT (U/L)	30	8–40
Inflammatory markers		
ESR (mm/h)	55	≤20
C‐Reactive Protein (mg/L)	20	0–10
Misc labs		
Total Protein (g/dl)	7.09	6.4–8.3
Albumin (g/dl)	2.80	3.5–5.0
Amylase (U/L)	37	30–110
PT INR	1.3	0.8–1.1
B12 Serum (ng/ml)	<159	200–900
RBC Folate (nmol/L)	1883.4	317–1422
Urine direct report		
pH	5.5	4.5–8
Sp. Gravity	1.02	1.005–1.025
Protein (mg/d)	+	≤150
Blood (RBCs)	+	≤3
Pus cells	6–8	0–4
Leucocytes (per hpf)	Numerous	2–5
RBCs (per hpf)	1–2	≤ 2
Stool direct report		
pH	Alkaline	Usually alkaline, (6.6)
Color	Yellowish brown	light to dark brown
Consistency	Semi‐solid	‒
RBCs (mg/gm)	1–2	<2–3
Cysts or Ova	None	None
Mucus	Present	Absent
Fat globules	Few	Fewer than 60 neutral fat globules
Immunoglobulins		
Anti‐transglutaminase IgA (U/ml)	9.125	<12
Anti‐transglutaminase IgG (U/ml)	3.495	<12

Kidneys, ureters, and bladder imaging (KUB) showed multiple cystic lesions on both kidneys of variable sizes but no other structural abnormality. Invasive procedures like esophagogastroduodenoscopy (EGD) and duodenal biopsy were done, which showed moderate pangastritis and fissured mucosa of the duodenum, termed as non‐specific chronic, active duodenitis on the biopsy report. A screening colonoscopy turned into a diagnostic procedure on viewing multiple small polyps, which were immediately resected and sent for histopathology.

**FIGURE 3:**
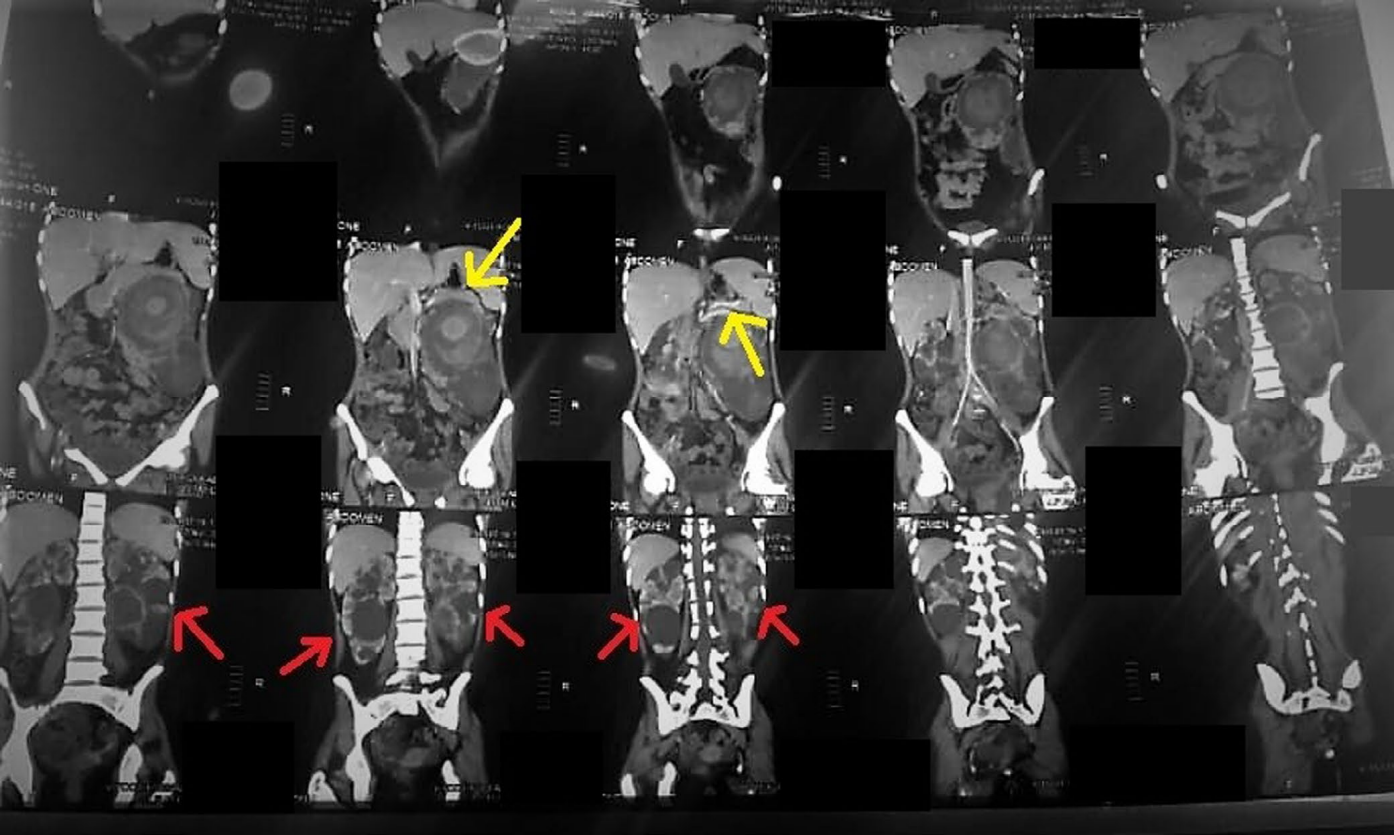
CT abdomen and pelvis shows swollen distal body and tail of pancreas (yellow), with few para‐aortic lymph nodes. Bilateral polycystic kidney disease (red) can also be seen

**FIGURE 4:**
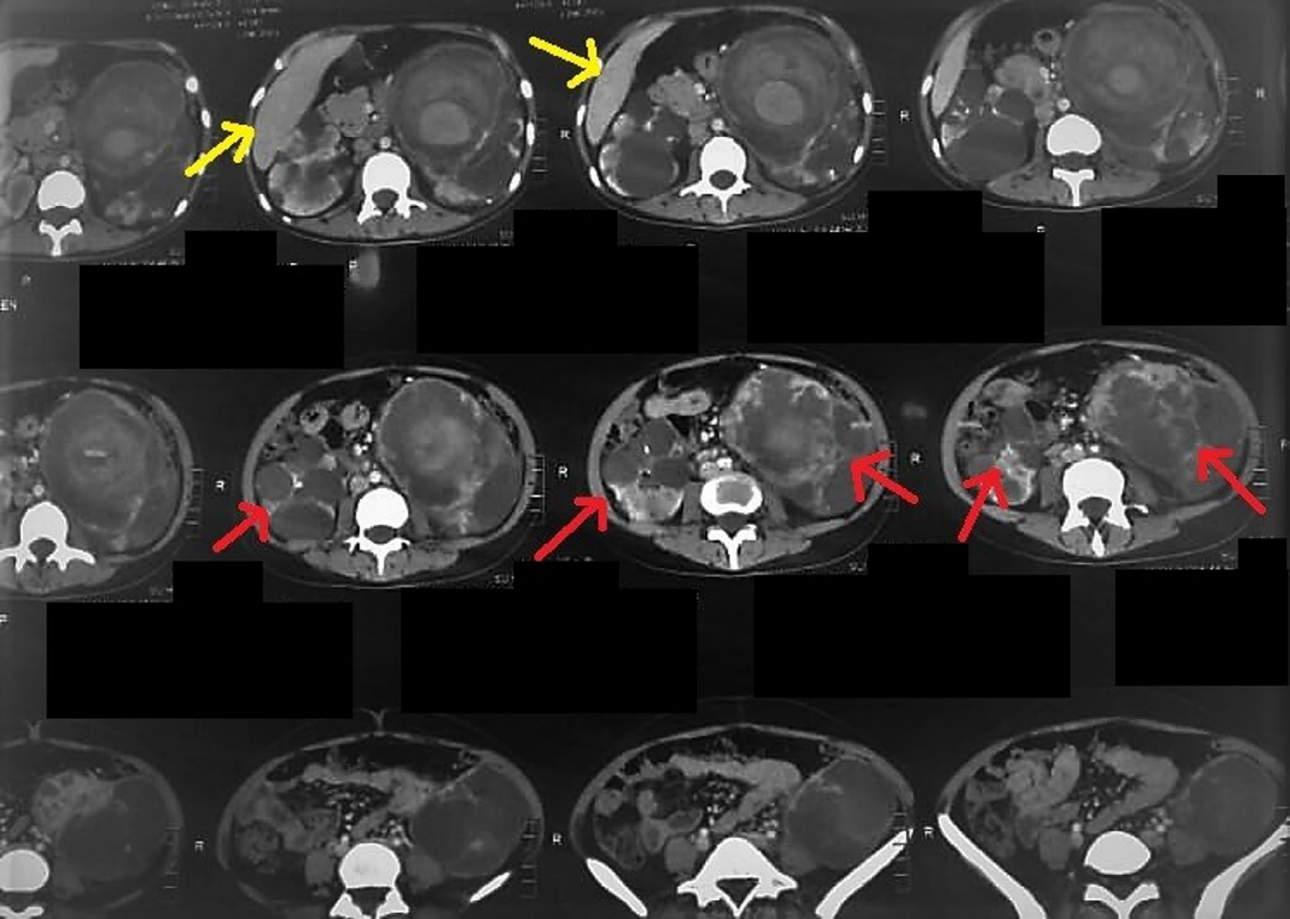
CT abdomen and pelvis shows bilateral renal cystic lesions, suggesting polycystic kidney disease (red). Additionally, large pseudoaneurysm formation on left side can be seen (yellow)

**FIGURE 5:**
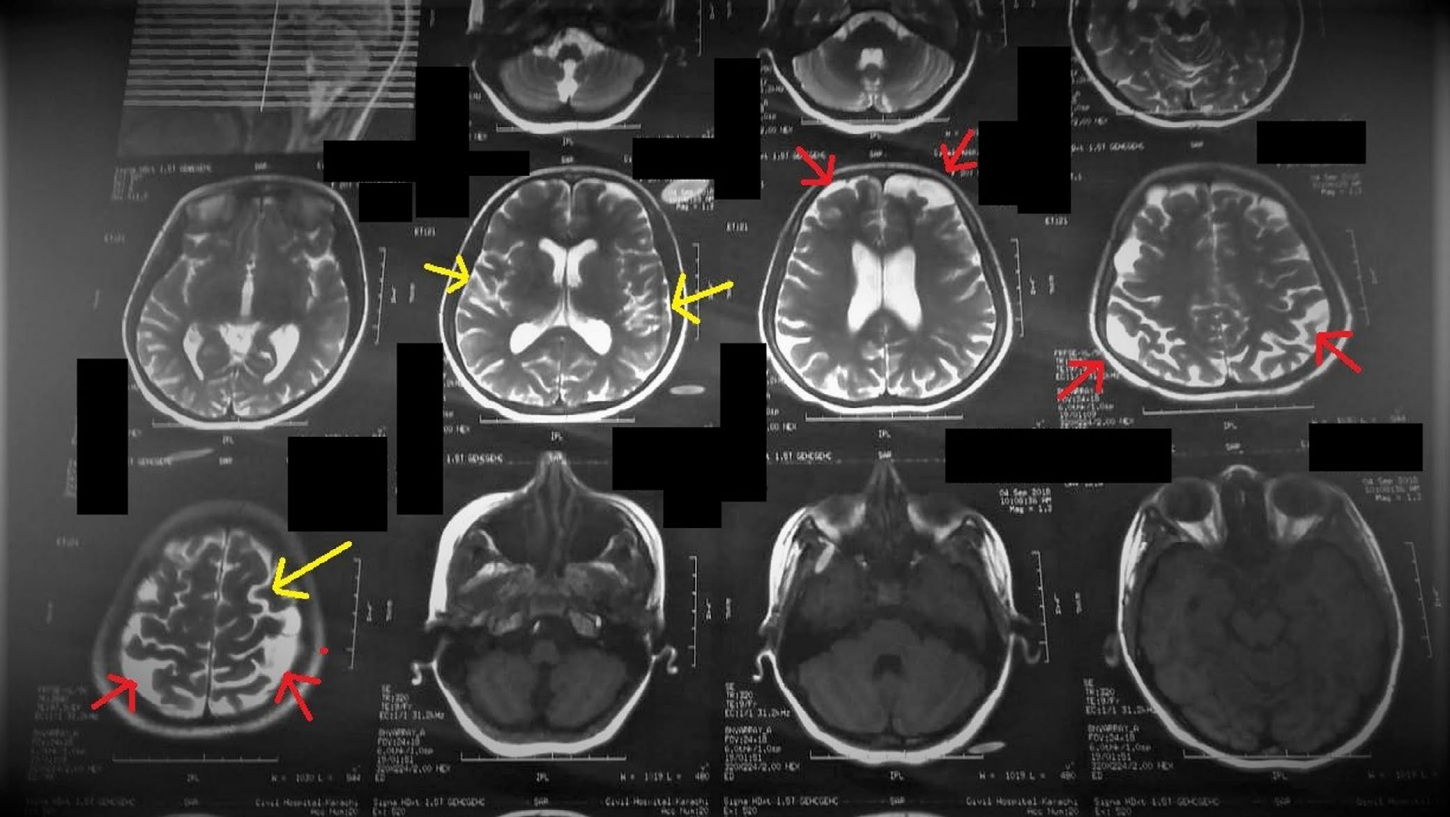
MRI of the brain shows multiple cerebral atrophy with an expansion of sulci, extra‐axial spaces, and thinning of gyri (yellow). Bilateral multifocal T2 and FLAIR hyperintense signals were seen in subcortical regions of fronto‐parietal lobes bilaterally (red)

On further evaluation a computerized tomographic (CT) imaging of the whole abdomen was done, documenting swollen distal body and tail of pancreas with tiny, non‐enhancing low‐density areas, along with a few visible para‐aortic lymph nodes, on the radiograph (Figure 3). Both kidneys confirmed the presence of cysts, earlier visualized on the KUB scan. The CT, however, reliably showed peripheral calcifications of the cysts (Figure 4). A relatively larger cyst on the left kidney had a pseudoaneurysm formed, and a further angiogram was suspicious of another small aneurysm of the distal segment of the left renal artery (Figure 5). Simultaneously, the echocardiogram revealed mild tricuspid and mitral regurgitations.

**FIGURE 6:**
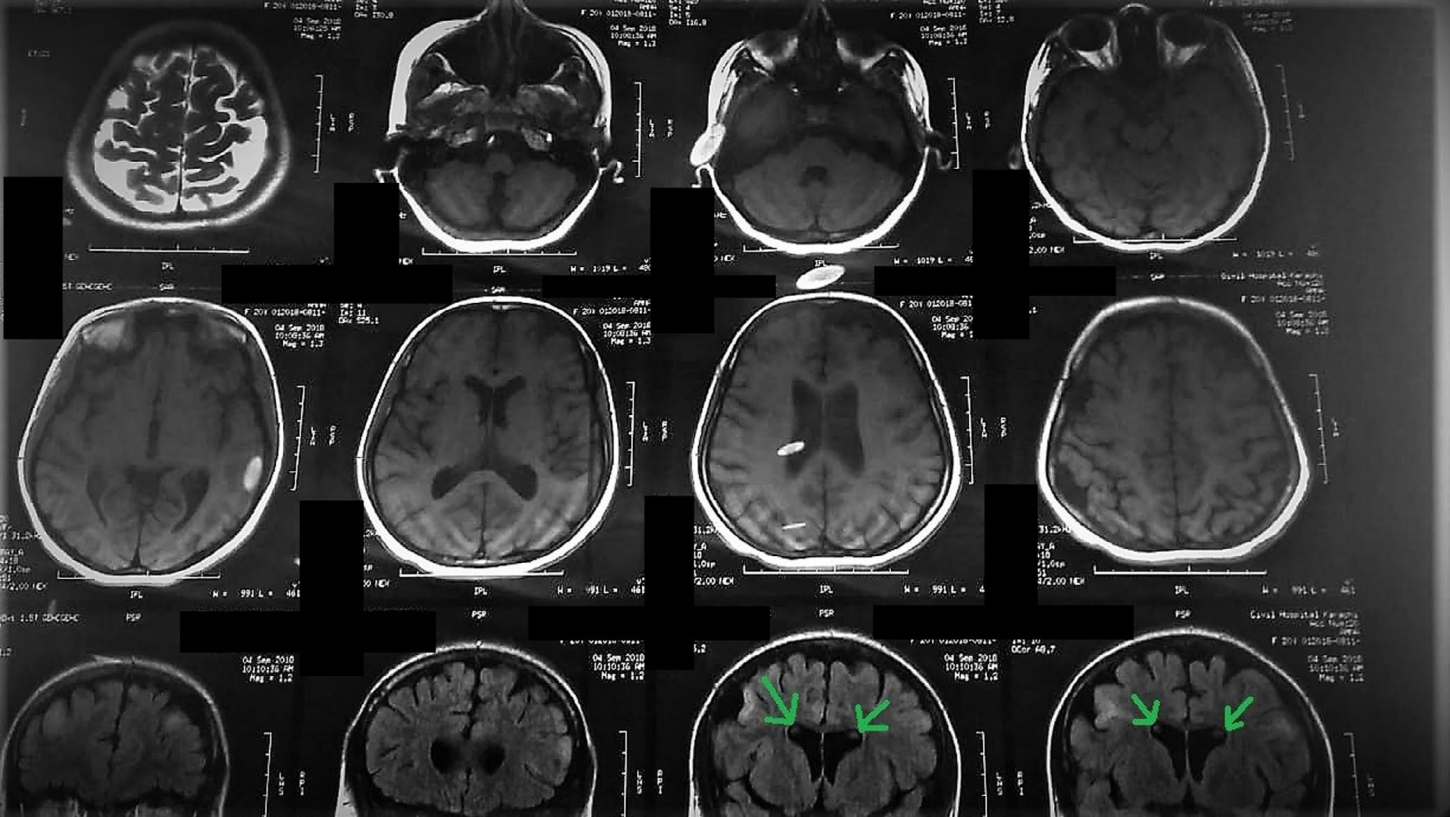
MRI of the brain (axial and coronal planes) shows subependymal nodules (green) are seen returning isohypointense signals onT2 and hyperintense on T1 without showing post‐contrast enhancement

**FIGURE 7:**
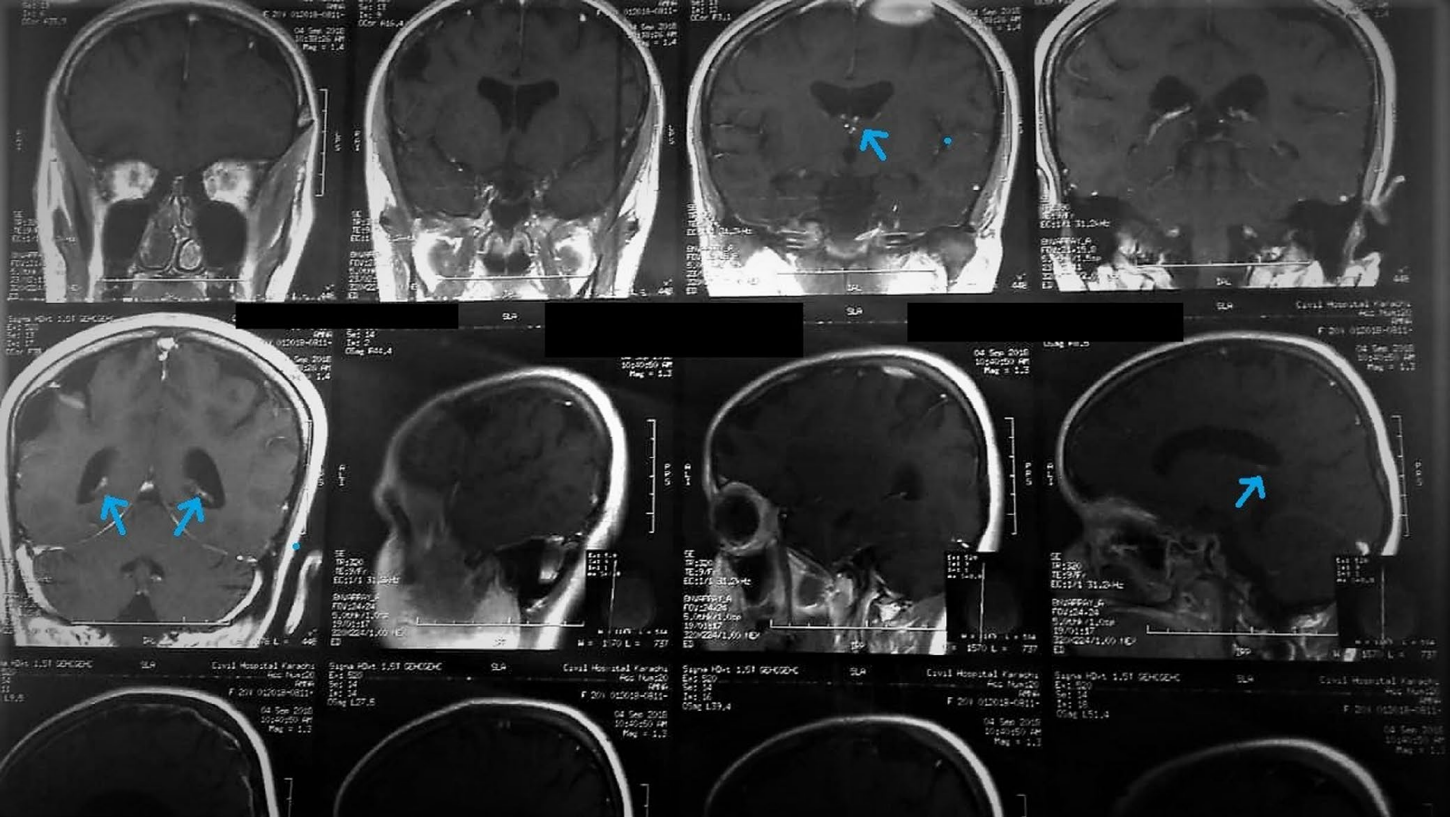
MRI of the brain (sagittal and coronal) shows subependymal nodules (blue) are seen returning isohypointense signals onT2 and hyperintense on T1 without showing post‐contrast enhancement

The final scan, that is, the magnetic resonance imaging of the brain, showed extensive cerebral atrophy with abnormal expansion of the sulci and the extra‐axial spaces (Figure 6). The gyri were notably shrunken with bilateral multifocal (T2) while fluid‐attenuated inversion recovery (FLAIR) showed hyperintense signals in the *subcortical* regions of the fronto‐parietal lobes. Furthermore, subependymal nodules were seen returning iso‐hypointense signals on (T2) and hyperintense on (T1) without showing post‐contrast enhancement (Figure 7). The magnetic resonance angiography and venography (MRA and MRV) reports showed no abnormal findings. On the basis of all investigations and physical assessments, a conclusion was drawn to label the patient as a case of TSC.

## 3 DISCUSSION

Tuberous sclerosis complex, or TSC, is a rare multisystem disease with significant genetic linkage of the autosomal dominant pattern. Cytogenetic studies have shown complete penetrance of the disease, meaning all individuals with genotype favoring TSC will present with some or all features of the disease, due to its variable expressivity. Two gene loci have been dubbed as hallmarks of genetic diversity in individuals clinically showing signs of TSC, that is, long arm of chromosome 9 (9q34) and short arm of chromosome 16 (16p13.3).^3^ These are, respectively, termed as TSC1, which encodes for hamartin while the latter labeled as TSC2 accountable for encoding tuberin. Initially, the diagnosis was based on a triad of clinical features, that is, epilepsy, problems with impaired intellect and angiofibromas. Currently, however, this classic disease presentation is only limited to a small proportion of patients.

Physicians have globally reported various CNS ailments in people with TSC, some of which present as epileptic seizures, poor cognitive, and other psychosocial skills often leading to the diagnosis of autistic spectrum disorders and attention‐deficit hyperactivity disorder (ADHD). Exact foci of tuberous insult are increasingly being identified with hefty advances in brain imaging. Characteristics lesions, such as cortical tubers, subependymal nodules, and giant cell tumors, have been successfully attributed to the differences in CNS involvement. The most common form of epileptic seizures in children with TSC is infantile spasms and is vigorously matched to future cognitive disabilities. The incidence of seizures in TSC has yet been confirmed to be 92%.^4^ TSC patients exhibit a range of intellectual ability and may require extensive investigation to rule out CNS damage.

A striking feature of TSC is angiomyolipoma (AML), a benign agglomerate of dysmorphic vasculature, smooth muscle cells, and adipose tissue.^5^ Their choice of accommodation had initially been limited to the renal tissue but recent documentations of hepatic findings have come to light. Renal complications, originating from these AMLs, have frequently given rise to tuberous sclerosis‐related deaths. An atypical complication is the formation of small or giant pseudoaneurysms in renal AMLs, which might require pertinent angioembolization to avoid any risk of rupture. Despite the different sites of AMLs, CT scan has been elected as the better choice of imaging than the MRI scans.

Since TSC massively employs multiple organs, the retina is frequently affected. Some evidence has been provided on a few TSC patients presenting with cardiac rhabdomyomas and pulmonary lymphangiomyomatosis, while others commonly presented with minor dental disorders, like gingival hyperplasia and enamel hypoplasia with its strong correlation to dental caries.^6^ Other rare features of TSC might include high arched palate, bifid uvula, cleft palate, delayed eruptions, and some reports have often stated diastemas in several patients.

Men with TSC might also present, rarely, with lymphangioleiomyomatosis (LAM), which are primarily cystic lesions reliably diagnosed on high‐resonance CT of the chest. Isolated cases of LAM differ from those associated with TSC due to earlier screening interventions in the latter. Hence, findings implying a positive diagnosis of LAM are usually incidental or identified in patients presenting most commonly with exertional dyspnea, or less commonly with spontaneous pneumothorax. In conclusion, ruling out LAM in cases suggestive of TSC might provide ample evidence to physicians for mapping out appropriate management plans.

In line with the aforementioned diagnostic criteria, our patient presented with three major features of the disease, that is, Shagreen patch, subependymal nodules, and cortical dysplasia along with few minor characteristics. These included bilateral, calcified renal cysts, and particularly of the left renal cyst exhibiting signs of pseudoaneurysm formation. Cortical dysplasia, as implied by generalized cerebral atrophy with wide sulci and extra‐axial spaces, thinned gyri, and a positive history of seizures since childhood, served as an influential discovery for reaching a diagnosis of TSC.

Approach to newly diagnosed TSC patients often involves a multidisciplinary action plan with reference to its unique manifestations, usually involving a direct consultation team of internal medicine specialists, neurosurgeons, and genetic counselors. Dermatologists with expertise in laser and dermabrasion techniques might be onboard depending on the extent of skin involvement. In patients with substantial cognitive dysfunction, treatment aims to develop behavior modification techniques and encourage independent thought processes for social‐skill building.

The prognosis of TSC is usually multifactorial, largely relying on the severity of presentation and extent of systems undergoing tuberous insult. Reports suggest a correlation between earlier diagnosis and low survival rates, with three‐quarters of infants expected to not surpass their twentieth decade of life whereas TSC cases diagnosed in adulthood usually depend on the extent of internal lesions and cerebral disfigurements, that is, atrophy and calcifications.

## 4 CONCLUSION

We presented the case of a 19‐year‐old woman with cutaneous and radiological features of TSC, which is a rare genetic neurocutaneous disorder. Radiological imaging plays a vital role in clinically diagnosing TSC, in addition to the history of skin lesions and convulsions. Although there is no cure, there is symptomatic therapy available. Individuals should be encouraged to join support groups around the country. Because TSC is a lifelong illness, a qualified doctor on a frequent basis should check patients. Moreover, in children with seizures, developmental delays, or mental disability, TSC should be ruled out in the diagnosis.

## CONFLICTS OF INTEREST

The authors declared no potential conflicts of interest with respect to the research, authorship, and/or publication of this article.

## ETHICAL APPROVAL

The study was conducted in accordance with the Declaration of Helsinki. The paper is exempt from ethics committee approval as only one case was reported.

## CONSENT

Written consent for publication was obtained from the patient.

## Supporting information

Supplementary Material

## Data Availability

The data that support the findings of this study are available from the "Sajjad Ali" upon reasonable request.
