# Supplementary material for: Incidental case finding of a 19‐year‐old woman with Tuberous Sclerosis Complex: A step‐wise Multidisciplinary approach
Source: Clin Case Rep. 2021 Oct 6;9(10):e04933. doi: 10.1002/ccr3.4933 (PMC8493370; doi:10.1002/ccr3.4933)
Supplement: Supplementary file 1 — Supplementary Material [file CCR3-9-e04933-s001.docx]

| **MAJOR FEATURES** | **MINOR FEATURES** |
| --- | --- |
| Hypomelanotic macules (>5-mm diameter) | “Confetti” skin lesions |
| Angiofibromas  or fibrous cephalic plaque | Dental enamel pits (>3) |
| Ungual fibromas | Intraoral fibromas |
| Shagreen patch | Retinal achromic patch |
| Multiple retinal hamartomas | Multiple renal cysts |
| Cortical dysplasias | Nonrenal hamartomas |
| Subependymal nodules |  |
| Subependymal giant cell astrocytoma |  |

**Supplementary 1:**  Clinical diagnosis criteria for TSC. Definite diagnosis must include two major features or one major feature with two minor features. Possible diagnosis must include either one major feature or 2 minor features.
